# Home-based postural exercise as an adjunct to duloxetine improves sleep quality, physical quality of life, and trunk mobility in fibromyalgia: a randomized controlled trial

**DOI:** 10.1007/s00296-026-06137-w

**Published:** 2026-05-22

**Authors:** Ceren Demir, Ömer Faruk Özçelep, Elife Ceyda Okumuş, Mehmet Canlı, Halil Alkan

**Affiliations:** 1 Department of Rheumatology, Aydın Atatürk State Hospital, Aydın, Turkey; 2 School of Physical Therapy and Rehabilitation, Kırşehir Ahi Evran University, Kırşehir, Turkey; 3 Department of Physiotherapy and Rehabilitation, Muş Alparslan University, Muş, Turkey

**Keywords:** Fibromyalgia, Exercise therapy, Duloxetine hydrochloride, Quality of life, Combined modality therapy

## Abstract

In fibromyalgia syndrome (FMS), current guidelines recommend combining pharmacological treatments such as duloxetine with exercise interventions. This randomized controlled trial aimed to evaluate the effectiveness of a home-based postural exercise program as an adjunct to pharmacological treatment in individuals with FMS. Fifty-one patients diagnosed with FMS according to the 2016 American College of Rheumatology criteria were randomly assigned to an exercise group (EG, *n* = 26) or a control group (CG, *n* = 25). Both groups received duloxetine therapy, while the EG additionally performed a home-based postural exercise program three times per week for four weeks. Outcomes included pain intensity, pressure pain threshold (PPT), sleep quality, anxiety and depression, quality of life, and trunk range of motion (ROM). Significant improvements over time were observed in pain intensity, psychological symptoms, sleep quality, quality of life, and PPT in both groups (*p* < 0.05). Significant group × time interactions favored the EG for sleep quality assessed using the Pittsburgh Sleep Quality Index (F = 11.07, *p* = 0.002, η²=0.184), the physical component of the Short Form-12 (F = 4.31, *p* = 0.043, η²=0.081), trunk flexion (F = 20.85, *p* < 0.001, η²=0.299), trunk extension (F = 8.34, *p* = 0.006, η²=0.146), and global trunk ROM (F = 35.10, *p* < 0.001, η²=0.417). No significant group × time interactions were observed for pain intensity, anxiety, depression, mental quality of life, or PPT (*p* > 0.05). Adding a home-based postural exercise program to pharmacological treatment may provide short-term additional benefits in sleep quality, physical quality of life, and spinal mobility in individuals with FMS; however, these findings should be interpreted cautiously due to the short intervention duration and absence of follow-up assessment.

## Introduction

Fibromyalgia syndrome (FMS) is a chronic musculoskeletal disorder primarily characterized by altered central pain processing, leading to generalized, diffuse, and non-inflammatory pain affecting multiple body regions [1]. In addition to widespread pain, FMS is defined by persistent fatigue, non-restorative sleep, multiple somatic symptoms, and cognitive dysfunction. Furthermore, pain and inflammatory mechanisms observed in inflammatory arthritis have been suggested to contribute to the development and clinical course of FMS [2]. These multidimensional symptoms substantially impair patients’ quality of life (QoL), daily functioning, and social participation. The global prevalence of FMS has been estimated at approximately 2.1%, with a markedly higher incidence among women [3].

In recent years, evidence-based guidelines have been developed to provide clinicians and patients with structured recommendations for managing the expanding spectrum of FMS treatment options [4]. Given the role of serotonin and norepinephrine in descending pain inhibitory pathways [5]. dysregulation within these neurotransmitter systems has been implicated in the pathophysiology of FMS [6]. Duloxetine, a serotonin–norepinephrine reuptake inhibitor, is therefore widely prescribed for symptom management in FMS. Although the recommended dose typically ranges from 30 to 60 mg/day, higher doses up to 120 mg/day are frequently used in clinical practice [7, 8]. However, duloxetine treatment may be associated with adverse effects such as nausea, anxiety, insomnia, and diarrhea, which may negatively influence treatment adherence and overall patient well-being [8, 9].

Current guidelines consistently recommend a multimodal approach combining pharmacological and non-pharmacological strategies in the management of FMS [4, 6, 10]. Among non-pharmacological interventions, physical exercise represents the cornerstone of treatment and has been shown to reduce pain severity, disease burden, anxiety and depressive symptoms, while improving QoL [11, 12]. Despite these well-documented benefits, adherence to structured and supervised exercise programs remains suboptimal in individuals with FMS. Barriers such as heightened pain sensitivity, fatigue, depressive symptoms, low self-efficacy, and logistical limitations significantly reduce participation rates compared to healthy populations [13]. Moreover, the global decline in physical activity levels—particularly following the COVID-19 pandemic has further emphasized the necessity of accessible and sustainable exercise models [14].

In this context, home-based exercise (HBE) programs have emerged as a feasible alternative that may enhance accessibility and long-term adherence by integrating physical activity into daily routines [15]. Recent systematic reviews and clinical studies have suggested that HBE interventions may improve pain, physical function, and QoL in individuals with FMS; however, the magnitude of benefit appears to vary according to exercise type, dose, duration, and adherence level [16–20]. Nevertheless, current evidence regarding the effectiveness of HBE in FMS remains inconclusive. While some studies report meaningful improvements in pain, depression, and QoL compared to non-exercising controls [15, 17], others demonstrate limited or inconsistent outcomes [18, 19]. Importantly, most previous investigations have evaluated HBE as a standalone intervention, without systematically examining its additive or synergistic effects when combined with standard pharmacological therapy.

To the best of our knowledge, no randomized controlled trial has evaluated the effectiveness of a structured home-based postural exercise (HBPE) program incorporating breathing techniques as an adjunct to duloxetine treatment in patients with FMS. This gap is clinically important, as postural correction and breathing regulation may influence musculoskeletal load, autonomic balance, and pain perception. Optimizing such non-pharmacological strategies alongside pharmacotherapy could enhance symptom control while potentially reducing treatment-related adverse effects and improving sustainability.

We hypothesized that integrating a HBPE program with duloxetine therapy would lead to greater improvements in pain, sleep quality, and QoL than pharmacological treatment alone, while potentially reducing adverse effects. Accordingly, this randomized controlled trial aimed to evaluate the effectiveness of HBPE as an adjunct to pharmacological treatment in individuals with FMS.

## Methods

### Study design and setting

This study was designed as a single-blind randomized controlled trial with concealed allocation and blinded outcome assessment. This randomized controlled trial was reported in accordance with the CONSORT reporting guideline for randomized trials, as recommended by the EQUATOR Network. The completed CONSORT checklist was submitted as a supplementary file (Supplementary File). Potential participants were screened for eligibility, and the study was carried out at the School of Physiotherapy and Rehabilitation at Kırşehir Ahi Evran University. All outcome measures were assessed by a blinded evaluator before the intervention and immediately after the 4-week treatment period.

Eligible participants were randomly assigned in a 1:1 ratio to two parallel groups: a control group (CG) receiving conventional treatment and an exercise group (EG) receiving home-based postural exercises in addition to conventional treatment. All interventions were administered by a physiotherapist with ten years of clinical experience who was aware of the group allocations. Outcome assessments were performed by a musculoskeletal physiotherapist with more than five years of clinical experience who was blinded to the participants’ group assignments. Ethical approval for the study was obtained from the Ethics Committee of Muş Alparslan University (Date: 03.06.2025; Approval No: 197135). The study was prospectively registered at ClinicalTrials.gov before participant enrollment (Registration No: NCT06738472; registration date: 25/01/2025; URL: https://clinicaltrials.gov/study/NCT06738472). The study was conducted in accordance with the principles of the Declaration of Helsinki, including the October 2024 revision. Written and verbal informed consent was obtained from all participants prior to participation.

### Participants

A total of 51 participants diagnosed with FMS were included in the study. Participants were recruited among patients who were newly diagnosed with FMS at the rheumatology clinic. The diagnosis of FMS was established by a specialist rheumatologist according to the 2016 American College of Rheumatology diagnostic criteria, which include the Widespread Pain Index and Symptom Severity Scale [21]. Individuals aged between 18 and 65 years, who had experienced widespread musculoskeletal pain for at least three months, and who were scheduled to begin pharmacological treatment for fibromyalgia were considered eligible for the study. In addition, participants were required to be able to understand and follow the study procedures and to participate in a HBPE program. All participants provided written and verbal informed consent prior to participation.

Patients were excluded if they had other rheumatologic diseases (such as rheumatoid arthritis, ankylosing spondylitis, or systemic lupus erythematosus), neurological disorders affecting movement or balance (e.g., stroke, Parkinson’s disease, or multiple sclerosis), severe psychiatric disorders, or cognitive impairments that could interfere with participation in the study. Individuals with serious cardiovascular, pulmonary, or metabolic diseases that could contraindicate exercise were also excluded. Additional exclusion criteria included pregnancy, a history of major surgery or trauma within the last six months, and current participation in a structured physiotherapy or exercise program for FMS. Patients who were unwilling or unable to comply with the study protocol were also excluded from the study.

### Interventions

#### Conventional treatment

All participants received conventional pharmacological treatment in accordance with the recommendations of the European League Against Rheumatism (EULAR) guidelines for FMS management. Duloxetine therapy was initiated at a dose of 20–30 mg/day, and the dosage was gradually titrated based on patient tolerability. The dose could be increased in increments of 20 mg every few weeks, up to a maximum of 60 mg/day, which has been reported as the average effective dose in clinical trials. Dose adjustments were individualized according to patient characteristics and treatment response, as lower doses such as 30 mg/day have also been shown to provide clinically meaningful benefits in some patients [9].

Evidence from previous studies indicates that duloxetine is more effective than placebo in reducing pain severity associated with fibromyalgia, both in short-term (≤ 12 weeks) and longer-term (≤ 28 weeks) treatment periods. Therefore, duloxetine therapy was administered to participants in both groups as the conventional pharmacological treatment throughout the study period [9].

### Home-based postural exercises

Participants in the exercise group received a HBPE program in addition to conventional pharmacological treatment. The exercise program consisted of four exercises targeting spinal mobility, postural control, and core muscle activation: the Cat–Camel exercise, Bird–Dog exercise, back stretch exercise, and thoracic mobility exercise. Prior to the intervention, all exercises were individually demonstrated and explained by an experienced physiotherapist to ensure that participants performed them with correct technique. Additionally, the exercise program was provided to the participants in the form of an illustrated brochure to facilitate proper implementation at home. Participants were instructed to perform the exercises at home three days per week for a total duration of four weeks. Each exercise was performed for three sets of ten repetitions, with approximately 30–60 s of rest between sets. To enhance adherence and monitor compliance with the HBPE program, participants in the exercise group were regularly followed up via telephone throughout the intervention period. During these calls, they were reminded about the exercise protocol and provided with guidance when necessary [22]. Exercise adherence was monitored through these regular telephone follow-ups, and participants were required to complete at least 80% of the prescribed exercise sessions to be considered adherent to the intervention protocol.

### Outcome measurements

Prior to the intervention, demographic and clinical characteristics, including participants’ age, height, weight, and duration of disease, were recorded.

### Pain Intensity

Pain intensity was assessed using the Visual Analog Scale (VAS). Participants were asked to indicate their current level of pain on a 10-cm horizontal line, where 0 represented “no pain” and 10 represented “worst imaginable pain”. Assessments were performed prior to the intervention and immediately after the treatment period by a blinded evaluator. This method allowed for a quantitative measure of pain severity and has been widely used and validated in patients with FMS [23].

### Pressure pain threshold assessment

Pressure pain threshold (PPT) was evaluated using a digital algometer and recorded in kg/cm². In patients with FMS, 18 tender points were assessed, including 9 points on the right side and 9 points on the left side, ensuring bilateral symmetry. Pressure was applied gradually at each point until the participant reported the onset of pain. The mean PPT value across all 18 points was calculated for each participant [24].

### Quality of life

Health-related Qol was assessed using the Short Form-12 (SF-12) questionnaire, which evaluates both the physical (PCS) and mental (MCS) component scores of well-being. Each component is scored on a scale from 0 to 100, with higher scores indicating better health-related quality of life. Participants completed the questionnaire prior to the intervention and immediately after the treatment period, providing a standardized and validated measure of perceived health status in patients with FMS [25].

### Anxiety and depression

Anxiety and depression symptoms were evaluated using the Hospital Anxiety and Depression Scale (HADS). The scale consists of 14 items, with 7 items assessing anxiety (HADS-A) and 7 items assessing depression (HADS-D). Each item is scored from 0 to 3, resulting in a subscale score range of 0–21 for both anxiety and depression, with higher scores indicating greater symptom severity [26].

### Sleep quality

Sleep quality was evaluated using the Pittsburgh Sleep Quality Index (PSQI), a widely used self-report questionnaire that assesses sleep quality and disturbances over the past month. The PSQI consists of seven components: subjective sleep quality, sleep latency, sleep duration, habitual sleep efficiency, sleep disturbances, use of sleep medication, and daytime dysfunction. Each component is scored from 0 to 3, and the global PSQI score ranges from 0 to 21, with higher scores indicating poorer sleep quality [27].

### Range of motion

Joint range of motion (ROM) was measured using a manual goniometer by the same blinded evaluator to minimize measurement variability. Trunk flexion and trunk extension were assessed separately, and the values for each movement were recorded in degrees. To improve measurement consistency, each movement was measured three times, and the mean value was used for statistical analysis. In addition to the individual measurements, the sum of trunk flexion and extension was calculated and reported as global trunk ROM, providing an overall assessment of spinal mobility [28].

### Sample size

An a priori sample size calculation was performed using pain intensity assessed by VAS as the primary outcome measure. Because directly comparable studies evaluating home-based postural exercise as an adjunct to pharmacological treatment in FMS were not available, the expected effect size was estimated based on previous exercise intervention studies in FMS. Given the exploratory nature of the study, a large effect size was assumed (Cohen’s d = 0.80), with a two-tailed significance level of α = 0.05 and 80% statistical power. Accordingly, approximately 25 participants per group were required. Considering potential dropouts, a total sample size of at least 50 participants was targeted [29].

### Randomization and blinding

Participants were randomly assigned to either the control group or the exercise group using computer-generated randomization via Randomizer.org. Allocation was concealed, and the study evaluators were blinded to group assignments throughout the study. The physiotherapist administering the interventions was aware of the group allocations, but all outcome assessments were performed by evaluators who were blinded to the treatment groups, ensuring unbiased measurement of study outcomes.

### Statistical Analysis

All statistical analyses were performed using SPSS version 25.0 (IBM Corp., Armonk, NY, USA). The normality of continuous variables was assessed using visual methods, including histograms and probability plots, and the Shapiro–Wilk test. Descriptive statistics for normally distributed variables were presented as mean ± standard deviation (SD). Between-group comparisons of baseline demographic and clinical characteristics were performed using independent samples t-tests. Pain intensity measured using the VAS was considered the primary outcome. To evaluate within-group changes over time and between-group differences in change over time, a mixed-design repeated-measures ANOVA was used, with time as the within-subject factor and group as the between-subject factor. The group × time interaction was interpreted as the main indicator of differential treatment effects between groups. Effect sizes were reported as partial eta squared (η²). Statistical significance was set at *p* < 0.05.

## Results

**Fig. 1:**
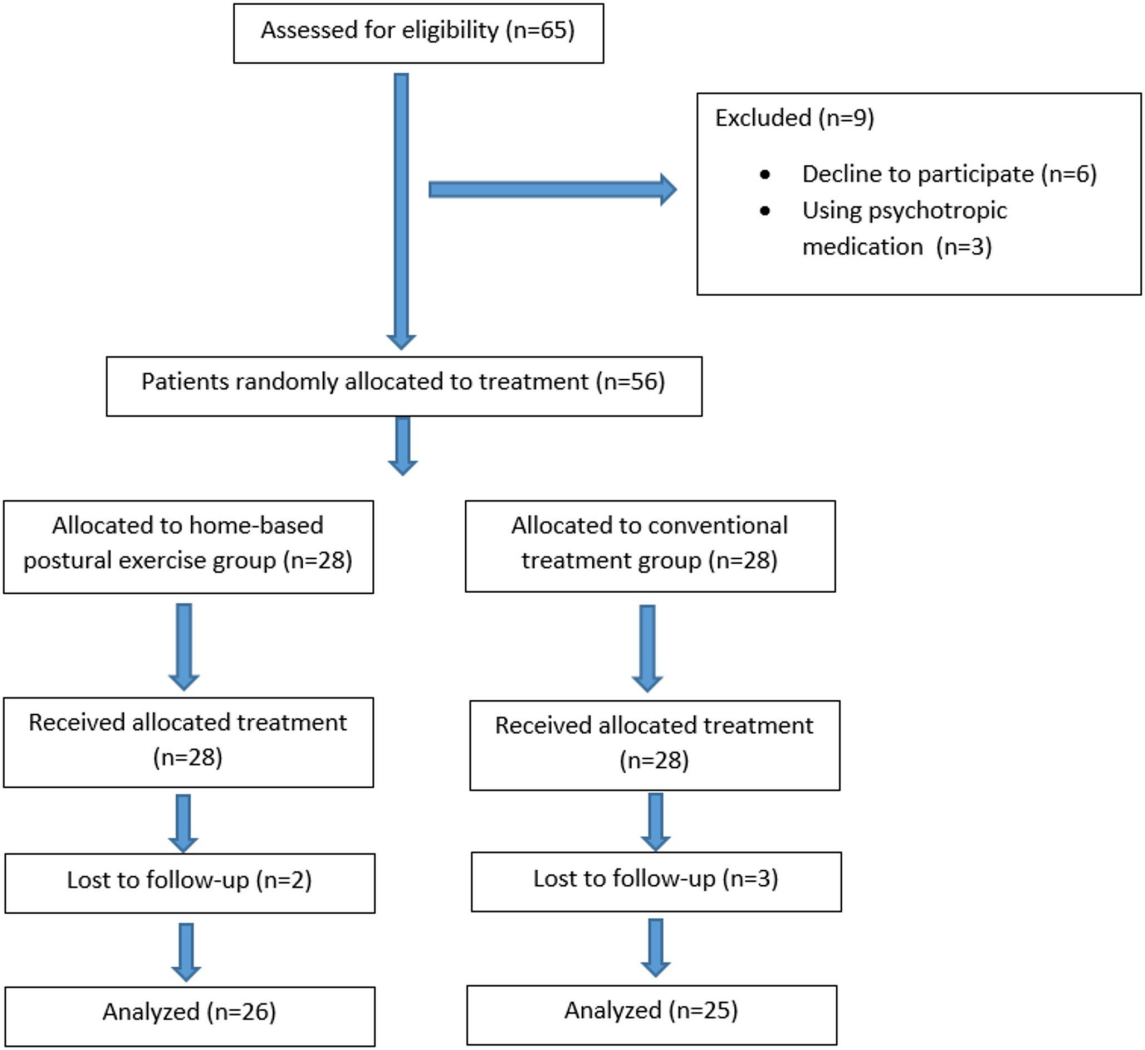
Flow chart of the study

A total of 65 individuals were initially screened for eligibility, of whom 9 were excluded because 6 declined to participate and 3 were utilizing psychotropic medications. Consequently, 56 patients were randomized and equally allocated to either the EG (*n* = 28) or the CG (*n* = 28). Throughout the intervention period, two participants from the EG and three from the CG were lost to follow-up. Final statistical analyses were performed with the remaining 26 participants in the EG and 25 in the CG (Fig. 1). Adherence to the HBPE program was satisfactory, as all participants in the exercise group completed at least 80% of the prescribed exercise sessions.

The comparisons of the descriptive characteristics of the groups included in the study are presented in Table 1. No statistically significant differences were observed between the groups in terms of their descriptive characteristics (*p* > 0.05). Accordingly, the groups included in the study were found to be comparable in terms of baseline characteristics.

**Table 1:** Comparison of descriptive characteristics of the groups

Variables	EG (*n* = 26)	CG (*n* = 25)	t	*p*
	Mean ± SD	Mean ± SD		
Age (years)	46 ± 6	46 ± 9	0.019	0.985
Height (cm)	1.64 ± 0.05	1.63 ± 0.05	0.904	0.370
Weight (kg)	77 ± 15	73 ± 12	0.988	0.328
BMI (kg/m^2^)	28.64 ± 4.94	27.81 ± 4.54	0.625	0.535
Disease Duration (months)	9 ± 6	10 ± 5	-0.419	0.677

*EG* Exercise group, *CG* Control group, *BMI* Body mass index, *SD* Standard Deviation

The comparison of pre- and post-treatment measurements between the EG and CG is presented in Table 2. According to the mixed-design repeated-measures ANOVA, significant main effects of time were observed for VAS, HADS-A, HADS-D, HADS total score, PSQI, SF-12 PCS, SF-12 MCS, PPT, and global ROM (*p* ≤ 0.011). However, the main effects of time for trunk flexion and trunk extension were not statistically significant (*p* = 0.061 and *p* = 0.154, respectively). Examination of the group × time interaction showed that the magnitude of change differed between groups for some outcomes. Significant group × time interactions were observed for PSQI (F = 11.07, *p* = 0.002, η²=0.184), SF-12 PCS (F = 4.31, *p* = 0.043, η²=0.081), trunk flexion (F = 20.85, *p* < 0.001, η²=0.299), trunk extension (F = 8.34, *p* = 0.006, η²=0.146), and global ROM (F = 35.10, *p* < 0.001, η²=0.417), with change scores indicating greater improvements in the EG. In contrast, the group × time interactions were not significant for VAS, HADS-A, HADS-D, HADS total score, SF-12 MCS, or PPT (*p* > 0.05), indicating comparable changes between groups for these outcomes. Overall, these findings suggest that although both groups improved over time in several outcomes, the EG achieved greater additional gains particularly in PSQI, SF-12 PCS, and trunk ROM parameters.

**Table 2:** Comparison of before and after-treatment test measurements between the EG and CG

Variables		EG (n=25)	CG (n=25)	Time	Group*Time	Between-Group Difference in Change Scores	η^2^
		Mean±SD	Mean±SD	p	F/p		
VAS (cm)	BT	8 ± 1	9 ± 1	< 0.001	0.26/0.612	0.37	0.005
	AT	5 ± 2	5 ± 3				
HADS-A (score)	BT	9 ± 5	9 ± 4	0.006	2.63/0.111	1.67	0.051
	AT	8 ± 3	7 ± 4				
HADS-D (score)	BT	9 ± 5	7 ± 6	0.001	0.01/0.942	–0.10	0.001
	AT	6 ± 3	5 ± 4				
HADS total score	BT	17 ± 8	16 ± 8	< 0.001	0.78/0.381	1.57	0.016
	AT	14 ± 5	12 ± 7				
PSQI (score)	BT	11 ± 4	10 ± 3	< 0.001	11.07/0.002	–1.52	0.184
	AT	7 ± 3	8 ± 3				
SF-12 (PCS)	BT	33.75 ± 8.70	28.62 ± 7.38	< 0.001	4.31/0.043	2.68	0.081
	AT	39.92 ± 10.44	32.11 ± 7.00				
SF-12 (MCS)	BT	38.37 ± 11.77	40.41 ± 8.93	< 0.001	0.03/0.862	–0.34	0.001
	AT	42.12 ± 10.57	44.51 ± 9.07				
PPT (kg/cm^2^)	BT	4.60 ± 2.02	4.83 ± 1.04	< 0.001	3.75/0.058	0.13	0.071
	AT	5.36 ± 2.03	5.46 ± 1.08				
Flexion (°)	BT	108 ± 9	112 ± 10	0.061	20.85/**<**0.001	6.61	0.299
	AT	113 ± 9	110 ± 12				
Extension (°)	BT	27 ± 8	26 ± 5	0.154	8.34/0.006	3.69	0.146
	AT	30 ± 8	25 ± 6				
Global ROM (°)	BT	135 ± 14	138 ± 11	0.011	35.10**/<0.001**	10.30	0.417
	AT	142 ± 13	135 ± 14				

EGExercise group, *CG* Control group, *BT* Before treatment, *AT* After treatment, *SD* Standard Deviation, *VAS* Visual Analogue Scale, *HADS* Hospital Anxiety and Depression Scale, *HADS-A* Hospital Anxiety and Depression Scale-Anxiety, Hospital Anxiety and Depression Scale-Depression, *PSQI* Pittsburgh Sleep Quality Index, *PPT* Pain pressure threshold, *ROM* Range of motion, η^2^ Effect size, p: Mixed design repeated measures ANOVA

## Discussion

The present randomized controlled trial investigated whether adding a structured HBPE program to duloxetine therapy provides additional benefits in individuals with FMS. The findings demonstrated that while both groups experienced significant improvements over time in pain intensity, psychological symptoms, sleep quality, QoL, and PPT, the addition of HBPE resulted in significantly greater improvements in sleep quality, the physical component of QoL, and trunk ROM. However, no additional benefits were observed for pain intensity, psychological symptoms, mental QoL, or PPT. These findings suggest that although pharmacological treatment contributes substantially to symptom improvement in FMS, the inclusion of targeted postural exercises may provide additional functional and sleep-related benefits.

The significant improvements observed in both groups in pain intensity and psychological symptoms are consistent with the known therapeutic effects of duloxetine in FMS. Duloxetine enhances descending inhibitory pain pathways through serotonin and norepinephrine reuptake inhibition, thereby modulating central pain processing mechanisms implicated in FMS pathophysiology [5, 6]. Previous systematic reviews and meta-analyses have demonstrated that duloxetine is effective in reducing pain severity and improving overall symptom burden in individuals with FMS [7, 10]. Recent pharmacological reviews also indicate that treatment response and tolerability may vary across individuals, supporting the need for individualized dosing and multimodal management strategies in FMS [6, 8]. Therefore, the time-related improvements observed in both groups in the present study may largely reflect the pharmacological effects of duloxetine on central pain modulation and associated symptoms.

Despite comparable improvements in pain outcomes, participants who performed the HBPE demonstrated significantly greater improvements in sleep quality compared with those receiving pharmacological treatment alone. Sleep disturbances represent a core feature of FMS and are strongly associated with central sensitization, pain amplification, and daytime fatigue [27]. Exercise is known to influence sleep through several physiological and neuroendocrine mechanisms, including regulation of circadian rhythms, modulation of autonomic nervous system activity, and increased endorphin release [10]. Moreover, postural exercises that incorporate controlled breathing and spinal mobility may promote parasympathetic activation and reduce muscular tension, thereby facilitating relaxation and sleep regulation. Previous studies have also reported improvements in sleep quality following exercise interventions in patients with FMS, supporting the role of physical activity in addressing sleep-related symptoms in this population [11, 12]. The present findings therefore reinforce the importance of integrating exercise-based strategies into multimodal treatment approaches for FMS.

One of the most prominent findings of this study was the substantial improvement in trunk ROM in the exercise group. Individuals with FMS frequently exhibit reduced physical performance, movement avoidance, and musculoskeletal stiffness, which may contribute to functional limitations [28]. Postural exercise programs that target spinal mobility and core stabilization may help improve neuromuscular coordination and reduce protective muscle guarding associated with chronic pain conditions. The exercises included in this study, such as Cat–Camel and Bird–Dog, are known to enhance spinal mobility and trunk muscle control. Consequently, regular performance of these exercises may contribute to improved flexibility and functional movement capacity. The large effect sizes observed for trunk range of motion parameters in the present study highlight the potential value of postural exercise interventions in improving functional outcomes in individuals with FMS.

Interestingly, the addition of exercise did not result in significantly greater improvements in pain intensity or PPT compared with pharmacological treatment alone. Several factors may explain this finding. First, the relatively short 4-week intervention period may have limited the potential for exercise-induced adaptations in central pain processing. Fibromyalgia is a chronic condition characterized by persistent symptoms, central sensitization, and fluctuating clinical presentations; therefore, a 4-week exercise intervention may be insufficient to induce clinically meaningful changes in pain modulation, pressure pain sensitivity, or broader symptom burden. Previous studies have suggested that exercise interventions lasting at least 8–12 weeks may be necessary to achieve more robust and clinically meaningful reductions in pain severity in FMS [12, 17]. Second, the early effects of pharmacological treatment may have overshadowed the additional effects of exercise on pain-related outcomes. Therefore, longer intervention periods and follow-up assessments are needed to determine whether HBPE provides additional benefits beyond pharmacological treatment for primary pain-related outcomes.

From a clinical perspective, the findings of the present study support the integration of accessible exercise strategies into FMS management. Adherence to structured exercise programs can be challenging for individuals with FMS due to fatigue, pain sensitivity, and psychological barriers [13]. Recent evidence further suggests that adherence to recommended exercise dosage is an important determinant of clinical outcomes in FMS, particularly for pain, sleep quality, fatigue, and overall health status [13]. HBE programs may therefore represent a feasible and practical approach that allows patients to integrate physical activity into their daily routines while minimizing barriers related to transportation and accessibility [15]. Consequently, combining pharmacological treatment with feasible home-based exercise interventions may contribute to more comprehensive and sustainable symptom management in FMS.

The present study has several strengths, including its randomized controlled design, blinded outcome assessment, and the use of standardized outcome measures. However, several limitations should also be considered. The relatively small sample size may limit the generalizability of the findings. In addition, the intervention period was limited to four weeks, which may not fully capture the long-term effects of exercise interventions in FMS. Given the chronic nature of FMS, this short duration may have been insufficient to determine the clinical relevance and sustainability of the observed changes, particularly for primary pain-related outcomes. Moreover, no follow-up assessment was conducted after the completion of the intervention; therefore, the long-term persistence of the observed improvements remains unclear. Another limitation is the absence of an attention-control condition. Telephone follow-up was provided only to participants in the exercise group to support adherence to the home-based program, which may have introduced differences in therapeutic attention between groups. Therefore, some of the observed benefits in the exercise group may partly reflect increased contact, motivation, or behavioral support rather than the specific physiological effects of the exercise program alone. Furthermore, although duloxetine dose titration was individualized according to tolerability and clinical response, detailed dose distribution between groups was not reported, which may limit the interpretation of whether pharmacological exposure was fully comparable between groups. Future studies including larger samples, longer intervention durations, attention-matched control conditions, standardized or more transparently reported pharmacological dosing, and follow-up assessments are needed to better understand the long-term effectiveness, sustainability, and specific treatment effects of HBPE in individuals with FMS.

## Conclusion

In conclusion, the findings of this randomized controlled trial suggest that adding a structured home-based postural exercise program to duloxetine therapy may provide short-term additional benefits in sleep quality, physical quality of life, and trunk mobility in individuals with FMS. However, no additional benefits were observed for pain intensity, psychological symptoms, mental quality of life, or pressure pain threshold compared with pharmacological treatment alone. Therefore, HBPE may be considered as an accessible adjunctive strategy within the multimodal management of FMS, particularly for improving functional and sleep-related outcomes. Nevertheless, given the short intervention duration, absence of follow-up assessment, relatively small sample size, lack of an attention-control condition, and limited information regarding duloxetine dose distribution, these findings should be interpreted with caution. Future studies with larger samples, longer intervention periods, attention-matched control conditions, standardized or transparently reported pharmacological dosing, and follow-up assessments are needed to confirm the long-term effectiveness and specific treatment effects of HBPE in this population.

## Data Availability

Raw data are available upon reasonable request from the corresponding author.

## Declarations

### Clinical trials registry

The study was registered on the Clinical Trials Registry (Registration number: NCT06738472)
